# Is Innate Memory a Double-Edge Sword in Alzheimer's Disease? A Reappraisal of New Concepts and Old Data

**DOI:** 10.3389/fimmu.2019.01768

**Published:** 2019-08-07

**Authors:** Francesca Salani, Valentina Sterbini, Eleonora Sacchinelli, Mariagrazia Garramone, Paola Bossù

**Affiliations:** Experimental Neuropsychobiology Lab, IRCCS Santa Lucia Foundation, Rome, Italy

**Keywords:** Alzheimer's disease, trained immunity, trained potentiation, trained tolerance, innate immune cells

## Abstract

An emergent concept in immunology suggests that innate immune system is capable to undergo non-specific long-term responses and to provide resistance by modifying the reactivity to sequential pathogen challenge. This phenomenon, named innate memory, involves epigenetic, and metabolic reprogramming of innate immune cells. Current literature shows that the innate memory process has a mainly beneficial role in host defense, but sometimes can exert detrimental effects, as common in many diseases. Alzheimer's disease (AD) is a progressive neurodegenerative disorder characterized by cognitive decline and dementia. Accumulating findings demonstrate that inflammation is involved in AD pathogenesis and progression and recent genetic and functional data confirm the driving role of the innate immune component in the disease. Furthermore, AD patients show high burden of the most relevant infectious agents and up-regulation of inflammatory features in their innate immune cells, including an activated, or “primed” status of myeloid phagocytic cells in both brain and periphery, resembling trained immunity conditions. Thus, it is conceivable that AD innate cells may be firstly involved in the attempt to resolve recurrent/persistent inflammation but then acquire a trained phenotype mostly unable to maintain the immune regulation, leaving uncontrolled or sometimes supporting the progression of neurodegeneration. The present review aims to summarize evidence evoking innate immune memory mechanisms in AD, and to interpret their potential role, either protective or harmful, in disease progression. A better understanding of such mechanisms will provide a fertile ground for development of novel diagnostic, and therapeutic pathways in AD cure.

## Introduction

Defense is a proper function of the organism that must protect itself from external or internal noxious agents. In vertebrates, as well as in invertebrates and plants, the first protection mechanism is orchestrated by the action of innate immune cells, which cooperate to eliminate hazardous stimula (1). Innate cells are able to fight a broad range of pathogens, although with non-specific responses. They were considered immediate mediators of host resistance and inflammation in contrast with the adaptive lymphocyte–dependent immune response that is antigen specific, and capable to provide lifelong protection against re-infection. Over time, it has been repeatedly observed that innate responses induced by exposure to one pathogen or vaccine could affect the following immune response to subsequent encounter of the same pathogen or a different one (2–4). So, the classical vision on innate immune system has been switched into the notion that it also holds a memory (5). Innate cells' memory is not related to gene rearrangements as in lymphocytes, but is a consequence of reprogramming based on gene transcription changes, epigenetic processes, and cellular metabolism (6) addressed to promote a protective response by increasing resistance to reinfection. However, under predisposing conditions, innate memory may endorse human diseases characterized by excessive inflammation, even causing a neuroinflammatory cycle in neuropathological conditions (7, 8). Inflammation is a driving force in Alzheimer's disease (AD), a progressive neurodegenerative disease leading to dementia. In particular, genomic studies have associated AD with dysregulated innate immune cells, and uncontrolled neuroinflammatory processes appear critical contributors in AD pathogenesis (9–11). Besides alterations in microglia -the brain resident innate immune cells-, blood borne cells, and peripheral inflammatory factors may play a pivotal role in AD pathogenesis and progression (12). However, the exact mechanisms by which innate cell response influences AD course is still elusive. Regardless the recent evidence suggesting a role for the innate memory status on neuroinflammation, and neurodegeneration in AD mice (13), no studies are directly addressed to evaluate innate memory pathways in clinical AD. Main goal of the present mini-review is to recapitulate the features of AD innate response consistent with a trained phenotype in patients, thus providing insights to better decipher the role of inflammation in the disease.

## Innate Immune Memory: Cells, Receptors and Mechanisms

Innate and adaptive immune responses are components of the host defense integrated system. While innate immunity is considered the first, fast, and non-specific line of defense, adaptive immunity is slower, antigen-specific, and endowed with a memory that makes future responses against the specific antigen more efficient. Innate immune system is composed of different cell types primarily including mononuclear phagocytes as monocytes, macrophages and dendritic cells, but also natural killers (NK), and innate lymphoid cells (ILCs); while B and T lymphocytes are components of the adaptive system. The primary innate immune cells of the brain are microglia, mononuclear phagocytes that act as sentinel of injuries like macrophages. In adult mice and humans, brain immune cell population is a combination of the resident microglia and other phagocytes, included infiltrated monocytes who differentiate in microglia-like cells, especially during chronic injuries. Microglia have different phenotypes: under normal conditions, when resting have ramified morphology, M1 indicates classically activated, and M2 alternatively activated microglia (14). Recently, a disease-associated microglia (DAM) type has been identified (15).

Recent findings have shown that all cells of the immune system are aware of the immunological experiences and stimuli they are exposed to and have a memory, non-specific in innate and specific in adaptive system. The concept of innate memory has been validated in many innate immune cells with mechanisms that involve epigenetic and metabolic cell remodeling, in contrast to gene rearrangements as in lymphocytes. This phenomenon called trained immunity is a specific immune program triggered by pathogen-derived molecules or other danger stimuli which confers to innate cells a memory. It is a long-lasting altered inflammatory activation, making cells able to respond to subsequent stimulations either more heavily or weakly. These two opposite activities, which are generally balanced for the well-being of the organism, are named trained immunity, and tolerance or, as more recently suggested, trained potentiation and trained tolerance (16).

Actually, memory mechanisms have been well-described for monocytes-macrophages (6) and NK (17, 18). Similarly, blood derived DC could be primed by infections, give protection against subsequent challenges showing epigenetic marks (19, 20), but their trained immunity features are not fully characterized. Moreover, recent results have shown in mice that microglia are also capable of being trained. In fact, peripherally administration of inflammatory stimuli can alter long-term microglia function, due to differential epigenetic reprogramming that persist during the time influencing neuropathology later in life (13).

As mentioned, epigenetic, and metabolic reprogramming characterize the immune training of innate cells. Changes in histone marks and chromatin architecture are related to an increased, or decreased metabolism and transcriptional processing (21). These mechanisms may be also systemically induced at the level of bone marrow progenitors, and maintained in the daughter cells (22, 23), determining a specific status of innate memory that in turn could influence the general inflammatory response.

Cells of the monocyte-macrophage lineage undergo long-term functional reprogramming following activation of pattern-recognition receptors (PRRs), that detect infectious pathogen-associated molecular patterns (PAMPs), but also non-infectious damage-associated molecular patterns (DAMPs). Like PAMPs, DAMPs might act as stimuli that activate cells of the innate immune system (24). Exposure to certain PAMPs, as those that bind to toll-like receptors (TLR), and Nod-like receptors (NLR), can confer a type of immunological memory to mononuclear phagocytes that depends on the type of pathogen and its dosage. Thus, activation of NPLR3 inflammasome by PAMPs or DAMPs, promoting the maturation and secretion of IL1β and IL-18, appears as a key mediator in the potentiation process of trained immunity induced by stimuli like BCG, β-glucan, and Western-type diet (7, 23, 25). At variance, the bacterial component lipopolysaccharide (LPS) binding to TLR-4, is known to induce tolerance (26). Another study demonstrated that trained potentiation and tolerance are two opposing functional programs, depending on the nature, and concentration of engaged PRRs. Hence, engagement of NLRs (NOD2 or NOD1 receptors) induces trained potentiation, which can vanish with smaller amounts of ligands, while the engagement of TLRs with high inflammatory doses of PRR ligands induces tolerance. Conversely, low concentrations of TLR ligands in monocytes reverse tolerance to potentiation, heightening the pro-inflammatory state (27).

At first stimulus, epigenetic reprogramming occurs and involves methylation or acetylation on N-terminal histone tails, like H3K4me, H3K9ac, and H3K27ac. For instance, after β-glucan treatment, monocytes show enrichment of H3K4me3 in promoters of genes encoding the pro-inflammatory cytokine TNF-α, IL-6, and IL-18 (28–30). After stimulation, some epigenetic marks are lost and cells maintain a low inflammatory gene expression. However, some enhancers, called latent enhancer, preserve a state of mono-methylation (H3K4me1) increasing accessibility of chromatin leading to a stronger response to subsequent stimuli (31). After a second stimulus, trained cells show higher inflammatory gene expression and acquire the epigenetic signature on their regulatory regions as H3K4me3, H3K4me1, and H3K4ac. After multiple inflammatory stimuli, cells could adopt a tolerance program consisting in the lack of responsiveness, resulting in low expression of pro-inflammatory cytokines, and acquiring epigenetic markers of transcriptional silencing, as in naïve state (32).

Transcriptional changes also reflect primarily metabolic activation. In monocytes, β-glucan-induced trained immunity leads changes in cellular metabolism from oxidative phosphorylation to aerobic glycolysis, increasing the ability of innate immune cells to respond to subsequent stimuli (33). In particular, the shift in metabolism leading to increased glycolysis is dependent on the activation of mammalian target of rapamycin (mTOR) through a dectin-1-Akt-HIF-1α (hypoxia-inducible factor-1α) pathway. In addition, the epigenetic changes observed in BCG-trained monocytes are dependent on the induction of the metabolic pathways: if glycolysis or glutaminolysis is inhibited, changes in H3K4me3, and H3K9me3 at promoter sites of IL-6 and TNF-α reverse, showing a link between these two regulatory cellular processes (34). Metabolic changes influence chromatin remodeling since epigenetic enzymes use small metabolic co-factors to perform their functions, therefore metabolic shift can cause altered epigenetic signature in immune cells (35). During immune memory, immune-metabolism and gene expression are linked by the epigenetic modifications.

## Alzheimer's Disease and Innate Immune Cells

The sporadic form of Alzheimer's disease (AD) is the most common type of dementia diagnosed in elderly and its cure or prevention still lacks effective treatments. Main AD histopathologic hallmarks are intracellular neurofibrillary tangles of hyper-phosphorylated tau protein and extracellular aggregates (plaques) of the misfolded amyloid-β (Aβ) peptides (36). Aβ plaques are surrounded by activated glial cells releasing inflammatory mediators, hence the sustained neuroinflammatory response has emerged as a third core pathological feature of AD (37), with an acknowledged role in disease pathogenesis and evolution (38, 39). Accumulation of Aβ, resulting from its imbalanced production and/or clearance, is widely considered a critical pathogenic event that induces microglial activation prompting to a local inflammatory process that in turn leads to amyloid plaque generation. Aβ production may be also connected with antimicrobial response, further strengthening the importance of immune system in AD (40). Genome wide association studies and integrative genomic analyses of brain transcriptomes confirm that myeloid cell-specific immune genes encoding for inflammatory factors and molecules involved in the clearance of misfolded proteins are risk factors for sporadic AD (10, 41–44).

The exact role of microglia underlying AD onset and progression is still unclear. Generally, microglia hold a protective role as they can sense and clear misfolded proteins, but in AD they may acquire a dysfunctional phenotype, secreting neurotoxic cytokines, and instigating a persistent inflammatory status (45, 46). It might occur in the earliest stages of the disease, while later on microglia and brain-invading monocytes would exert a prevalent beneficial function, triggering a resolution phase, possibly perturbed (47, 48). Accordingly, the DAM microglia, identified in the brain of AD mice and patients, seems to have the potential to restrict neurodegeneration (15). Overall, microglia reactions may be influenced by duration of activation, localization, involvement of other cell types, genetic susceptibility, aging, and disease progression (11), though a PET study suggests that the extent and dynamics of beneficial or detrimental microglia activation vary among patients, rather than depending on disease stages (49). Such view, in addition to explaining the trouble in decoding microglia role in AD, appears in agreement with the potential effects of innate memory response on microglia phenotype (13), which essentially depend on the previous history of exposure to priming factors in each individual.

Inflammation in both brain and periphery may be a very early event in the AD pathogenic course (37, 50–52). Microglia are able to sense inflammatory signaling molecules originating outside the brain. Peripheral inflammation, as well as activation of blood borne innate immune cells, appears to hold relevant disease-modifying functions in AD (50, 53, 54). As shown in animal models, blood-derived myeloid cells affect AD-like neurodegeneration with protective, causative, and/or reactive effects, apparently influenced by the disease stage (55–57). Thus, when evaluating the innate cell contribution to AD pathogenic pathways, both brain resident, and circulating innate immune myeloid cells should be taken into consideration. The latter could participate in the long-lasting dysregulated AD immune response by acting either directly when recruited to brain, or indirectly through the release of soluble mediators.

The most studied blood borne innate cell populations in AD are monocytes and macrophages, which enter the brain, and modulate pathology, although with controversial roles (58–60). Regarding other types of myeloid cells, an involvement in AD neuroinflammation and neurodegeneration is proposed for dendritic cells (DCs) (61, 62) and for neutrophils, which could cross into the brain parenchyma, and contribute to neuronal damage and cognitive decline (63). Eventually, NK may have some relevance in AD, but their contribution needs to be further clarified (64, 65).

Given this scenario, wherein AD progression is likely driven by an interplay of innate cell types, the memory status and capability of these effector cells to respond to neurodegenerative conditions by means of neuroinflammatory modulation is of primary importance to delineate the neuroprotective or damaging consequences of their activities.

## Evidence of Innate Memory Pathways in AD

Systemic infections, aging and chronic inflammatory conditions drive innate immune cells to undergo reshaping and they are all well-recognized risk factors of AD. In particular, Herpes simplex virus type 1, Cyto-megalovirus, Chlamydophila pneumoniae, spirochetes, Helicobacter pylori, and periodontal pathogens have been associated with AD, and cognitive decline (66–68). Some of them have been found in AD patients' brain, listed as causative factors of AD inflammatory pathways and implicated in disease pathogenesis (69–71). Emergent studies reported a microbial dysbiosis in both AD animal models and patients, which could affect Aβ amyloidosis and host innate immunity mechanisms (72), leading to a peripheral inflammatory state (73, 74). These results support the inflammatory-infectious theory of AD (75, 76). Accordingly, elevated plasma levels of LPS were previously described in AD patients (77) and recently found to progressively accumulate in AD brain in association with neuropathology, affecting gene expression (78). As reported, infectious stimuli, including bacterial or fungal cells and their components, as well as viruses, are considered potent inducers of innate immune memory, thus the role played by infectious agents in AD advocates for a reshaping of innate cell response in patients. In addition, complex human diseases with chronic inflammatory components in their etiologies, such as atherosclerosis, arthritis, diabetes and obesity, are predisposing factors for subsequent dementia (79), strengthening the link between a persistent activation of innate response and AD. Finally, aging, the most important risk factor for AD, causes inflammaging (80), a low-grade inflammation characterized by up-regulation of pro-inflammatory mediators and increased response of innate cells, possibly contributing to the pathogenesis of AD (81).

In keeping with a potentiated trained immunity status of AD innate cells, several authors have reported elevated levels of circulating pro-inflammatory cytokines and amplified inflammatory response of blood cells in AD patients. Despite some inconsistent results, meta-analysis studies confirm an overall trend of increased pro-inflammatory cytokines in AD (82, 83), and higher levels of cytokines were observed in patients with early or mild forms of AD (84, 85). Similarly, peripheral innate cells of AD patients show a “primed” state and the percentage of peripheral monocytes producing pro-inflammatory cytokines increases early in AD (86). Furthermore, AD monocytes and DCs show enhanced inflammatory phenotype in relation with symptom severity (87–89). When the disease progresses to a more severe condition, AD patients show a decrease in the levels of inflammatory markers (90, 91). Thus, at variance with the earliest phases, the depressed innate response observed in late stages of AD might reflect a condition of innate tolerance following repeated and unresolved innate stimulations.

AD microglia also show modifications that remind an adaptive process mediated by trained immunity (92). In fact, in the aging brain and more evidently in AD patients, microglia appear primed: they are activated, produce increased amounts of pro-inflammatory mediators and are more susceptible to central damage after peripheral insults (93, 94). In addition, microglia activation can be suppressed by epigenetic modulation and epigenetic changes occurring during AD may prime microglia for a later transition to the DAM phenotype (15, 95), suggesting that epigenetic mechanisms are important in microglial priming. This picture fits well with what described in an AD experimental model, where after repeated systemic challenges mimicking bacterial invasion, microglia are epigenetically reprogrammed and, depending on the persistence of triggering, they undergo either a potentiated trained immunity resulting in amplification of pro-inflammatory mediator release or an acquisition of trained tolerance characteristics, both shaping neuropathology (13).

Converging evidence point to PRR activation, histone modifications and metabolic changes of innate cells in AD progression. Specifically, misfolded proteins like Aβ are able to bind PRRs expressed by microglia and other innate myeloid cells and exert cell activation with resultant release of inflammatory mediators, ultimately contributing to disease progression and severity. Aβ, both in its soluble and fibrillary form, is able to bind a variety of receptor molecules promoting inflammation, including CD14, CD36, and TLRs (96, 97). It can also act as a DAMP and activate the inflammasome NALP3 that leads to the release of the active pro-inflammatory cytokines IL-1β and IL-18, key components of the innate immune reaction observed in AD mice brain (98). Consistently, NLRP3 inflammasome activation occurs in the brains of AD patients, and contributes to pathology in AD mice (99).

In addition to resulting activated through PRRs engagement, AD innate cells show changes in cell transcription, epigenetic, and metabolic modifications that are mostly consistent with molecular mechanisms underlying trained immunity. Increased immune activity during AD neurodegeneration appears linked to aging and environmental-driven epigenomic alteration (100) and early epigenetic changes have been described in AD patients that may contribute to disease pathology (101). For instance, a mis-localization of the epigenetic molecule H3K4me3 between the cell nucleus and the cytoplasm has been reported in early AD (102), though its functional role is still unclear. In AD-mouse model of neurodegeneration, transcriptional and epigenetic changes (including H3K4me3 and H3K27ac) have emerged by profiling chromatin state across early and late AD pathology. Changes in immune genes and regulatory regions during AD-like neurodegeneration in mouse have been found, with strong human-mouse conservation of gene expression, and epigenomic signatures, especially in innate immune cells (103). Finally, AD innate cells are likely to undergo a raise of aerobic glycolysis, the metabolic driver of trained immunity. In fact, mTOR signaling is early increased in AD animal models (104), while TREM2-deficient mice with AD-like pathology show defective mTOR signaling, which affects ATP levels and biosynthetic pathways (105). Finally, in AD mouse model, mTOR activation and HIF-1α signaling, possibly mediated by Aβ-induced epigenetic microglial reprogramming, appear unfavorable to AD pathology (13).

## Potential Role of Innate Memory in AD and Concluding Remarks

Memory status of innate immune cells is governed by the type and concentration of ligand encountered and largely depends on the personal history of exposure to damaging agents during life. Although innate memory's role is mainly protective, it may in some cases influence the course of diseases, especially in those pathological conditions having chronic inflammation as hallmark, like AD. Likely due to many different circumstances, as enhanced infectious burden, microbial dysbiosis and persistence of endogenous misfolded proteins, AD patients show evidence of innate immunity chronic activation that could lead to maladaptive responses, mainly exacerbating damaging mechanisms in the brain.

**Figure 1:**
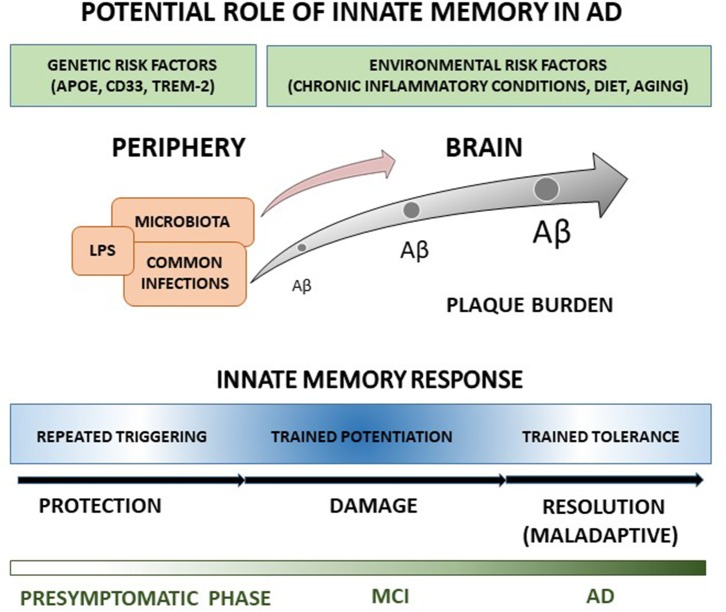
The cartoon recapitulates our view of the potential role in AD of innate memory processes occurring during disease progression. A detailed explanation of the drawing is reported in the text.

Regardless no studies have been addressed yet at evaluating innate memory responses in clinical AD, features of innate immune response in patients and animal models suggest that AD innate cells, including microglia, monocytes and DCs, may undergo a long-term functional reprogramming characterized by both potentiated and tolerant responses, possibly contributing to disease development. Under this view, we elaborated a schematic representation (Figure 1) according to which peripheral and cerebral innate cells of AD patients hold a memory of past stimulations that alters brain immune responses to Aβ, which in turn accumulates, contributing to the central propagation of pathological changes, and progressive clinical symptoms of AD. Since individuals are exposed to a multiplicity of different threatening and pathogenic agents during their life, a large heterogeneity in innate memory response is expected, in agreement with the high variation of microglia features, and dissimilarities in disease severity and progression among patients. Specifically, trained immunity could have potentiation features at pre-symptomatic and early times of AD progression, being characterized by enhanced release of pro-inflammatory cytokines, increased Aβ production and damaging consequences on affected brain. In contrast, at AD later stages, high concentration of persistent stimulus (e.g., Aβ) could switch the immune response toward trained tolerance. The prolonged exposure to stimulus could bring to desensitization with reduced production of inflammatory cytokines, shifting toward maintenance, and repair activities, though possibly inefficacious because of the maladaptive nature of the response.

Future studies should investigate the innate memory status and its specific molecular mechanisms in *in vitro* models of both peripheral and brain innate cells of AD patients, especially in relation to disease progression. Accordingly, considering the availability of inhibitors of immune-metabolic and epigenetic pathways leading to trained immunity (7), potential new strategies for AD treatment could be envisaged. Overall, a better knowledge of innate memory processes in AD could help in deciphering patients' inflammatory mechanisms underlying pathophysiology and thus facilitating the design of personalized treatments.

## Author Contributions

FS and PB made a substantial, direct, and intellectual contribution to the article designing and wrote the draft. VS, ES, and MG contributed to writing and revising the article. All authors have reviewed and approved the final version of the manuscript for publication.

### Conflict of Interest Statement

The authors declare that the research was conducted in the absence of any commercial or financial relationships that could be construed as a potential conflict of interest.
